# Traumatic Mallet Hallux: Two Case Reports With Distinct Treatment Approaches

**DOI:** 10.7759/cureus.100804

**Published:** 2026-01-05

**Authors:** Mafalda Reis, Filipe Maçães, Belmiro Alves, Margarida Gomes, Sara Macedo

**Affiliations:** 1 Orthopaedics and Traumatology, Unidade Local de Saúde de Gaia e Espinho, Vila Nova de Gaia, PRT; 2 Orthopaedics and Traumatology, Unidade Local de Saúde do Nordeste, Macedo de Cavaleiros, PRT

**Keywords:** avulsion lesion, extensor hallucis longus avulsion, hallux interphalangeal joint fracture, mallet hallux, toe fractures, traumatic toe injury

## Abstract

Traumatic mallet hallux, an avulsion injury of the extensor hallucis longus tendon or its bony insertion, is rare and often underdiagnosed, with no established treatment guidelines. We report two cases illustrating distinct management approaches based on fragment size and interphalangeal (IP) joint congruency. The first case - a nondisplaced avulsion fracture involving 30% of the articular surface - was successfully treated conservatively with extension splinting, achieving full recovery at three months. The second case - a 50% articular fracture with volar subluxation - required open reduction and internal fixation with a single screw, allowing early mobilization and complete functional restoration by three months. These cases highlight the importance of individualized treatment, where conservative management is suitable for stable injuries while surgery is indicated for displacement or joint incongruence.

## Introduction

Mallet hallux, or a traumatic mallet toe of the hallux, is the avulsion of the extensor hallucis longus (EHL) tendon, which is rare. Mallet toe has been extensively reported in lesser toe fingers and, even in lesser fingers, is rare as a traumatic cause [1]. In that regard, there are few studies, case reports, or even series of these patients, and there are no guidelines for their treatment.

As the mallet finger of the hand, the lesion can be a bony avulsion or a pure tendinous rupture [2]. Some authors described that it usually occurs due to blunt trauma with forced flexion of the interphalangeal (IP) joint of the hallux [3], for example, a fall from the stairs with the foot being caught on the step.

Regarding clinical findings, patients generally complain of pain, swelling of the greater toe, with or without ecchymosis. Physical examination shows a flexion deformity of the IP joint, which is passively but not actively correctable [4].

Concerning treatment, there is no consensus about the criteria to treat conservatively or surgically. Some authors make a parallelism with mallet finger of the hand criteria, reporting articular subluxation and more than 30% of joint involvement [2]. Regarding the type of procedure, there have been some types described, and none is consensual.

We present two cases of this lesion and report a different treatment for each one according to their particularities.

## Case presentation

Case 1

**Figure 1:**
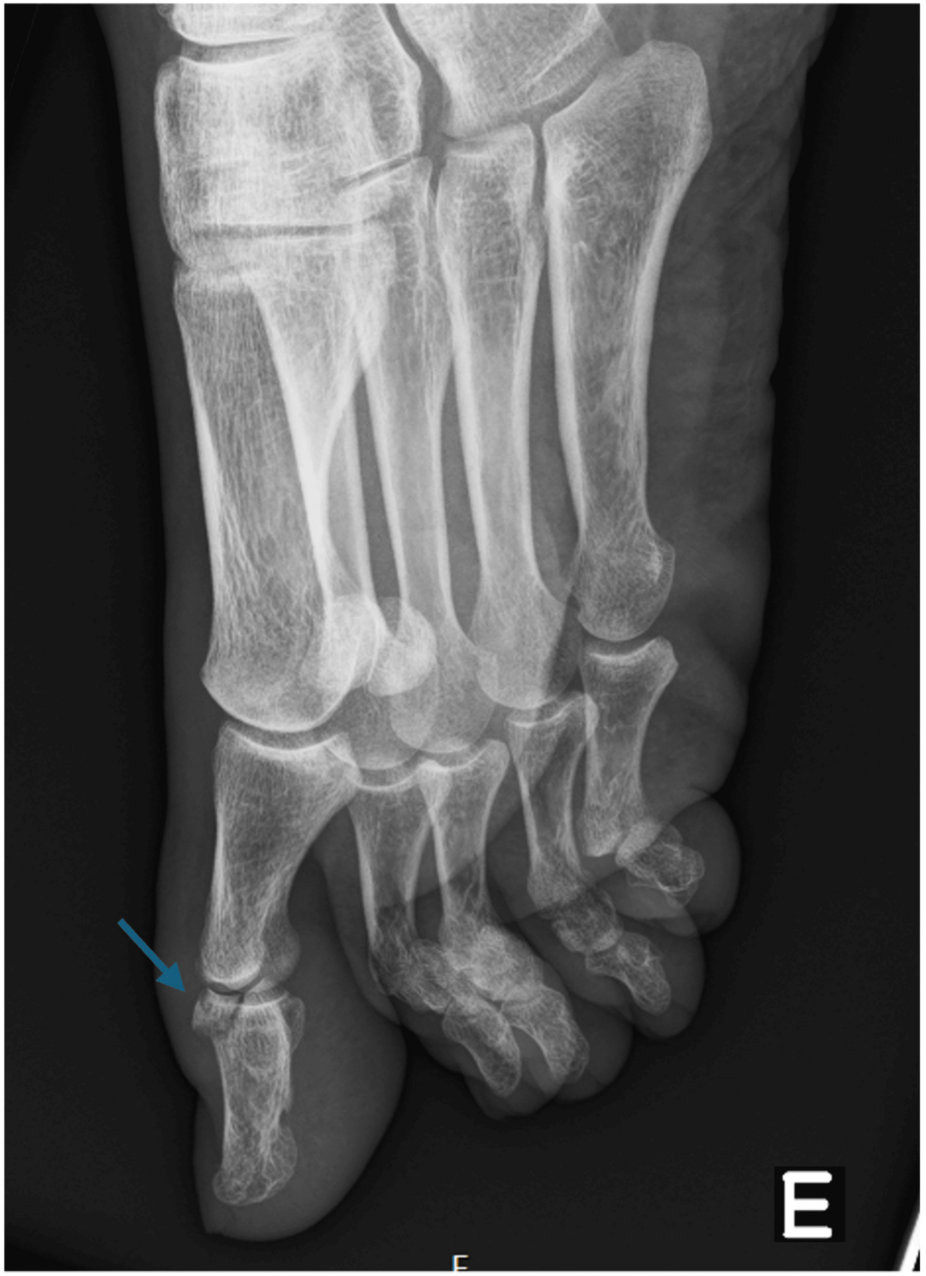
True lateral view of the hallux taken day 0 in the emergency department, showing the fracture (blue arrow) with minimal displacement and no subluxation.

A 42-year-old man suffered a fall, stubbing his left toe in hyperflexion. He presented to the emergency department on the same day, complaining of pain and incapacity for hallux weight bearing. The hallux showed an extensive dorsal equimosis and an inability to extend the IP joint. X-rays revealed a dorsal fracture of the distal phalanx involving 30% of the IP joint but without displacement or joint subluxation (Fig. 1).

**Figure 2:**
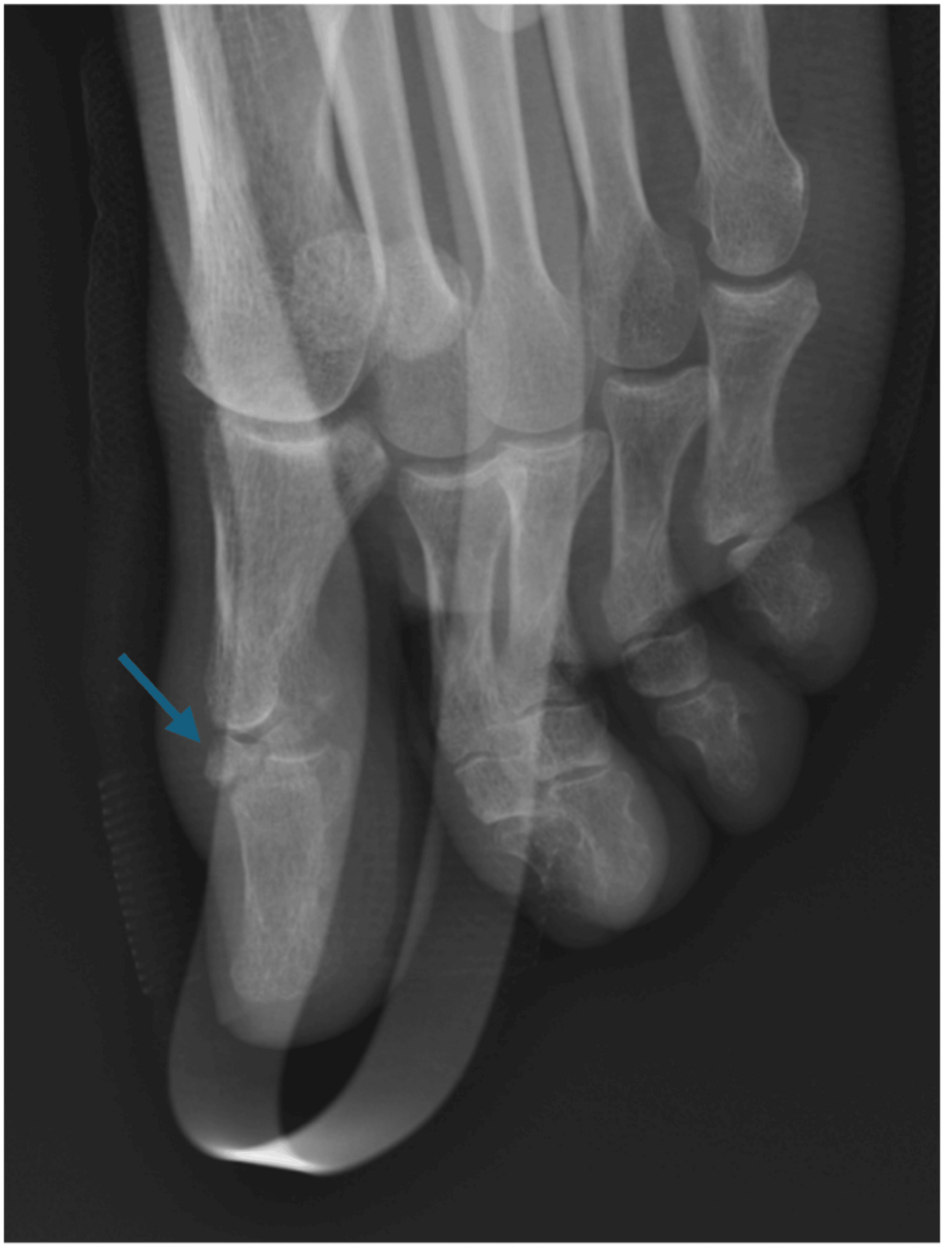
Lateral view of the hallux eight weeks after the injury. Bony fragment (blue arrow) remains in its place and shows some bone healing.

The IP joint was immobilized with a dorsal splint in extension for eight weeks, and a heel weight-bearing wedge shoe was recommended. The patient underwent follow-up X-rays, showing acceptable bone healing at eight weeks (Fig. 2). After 12 months of follow-up, he had no impairment and a full active range of motion (ROM) with no flexion deformity or pain. 

Case 2

**Figure 3:**
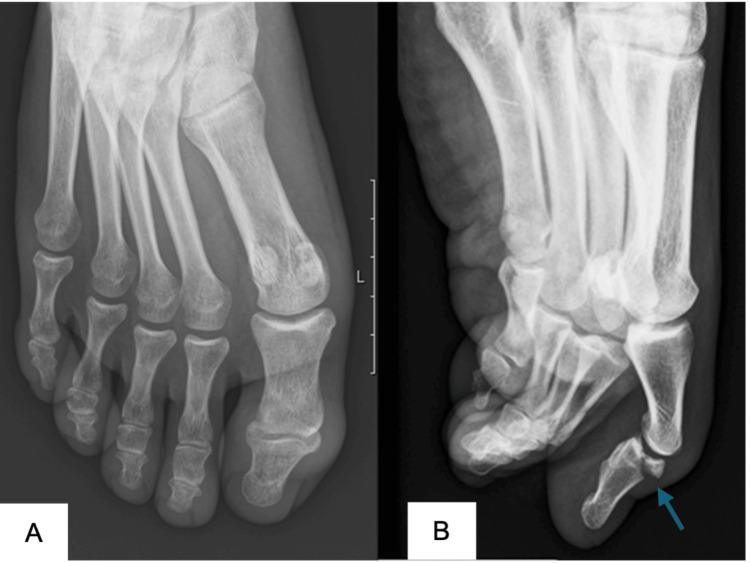
Front (A) and lateral (B) views of the hallux taken in the emergency department. It reveals interphalangeal (IP) subluxation and a 50% joint involving displaced bone fragment (blue arrow).

A 29-year-old woman fell from the stairs with her toe in hyperextension. She presented to the emergency department complaining of pain and inability to bear weight in her hallux. The image showed a dorsal fracture of the distal phalanx, involving 50% of the IP joint and volar subluxation of the IP joint (Fig. 3). We tried closed reduction unsuccessfully. Then, we proceeded to surgical treatment with open reduction and a 1.7 mm single cortex screw fixation. No post-operative immobilization was used, but a Barouk shoe was recommended for weight bearing. 

**Figure 4:**
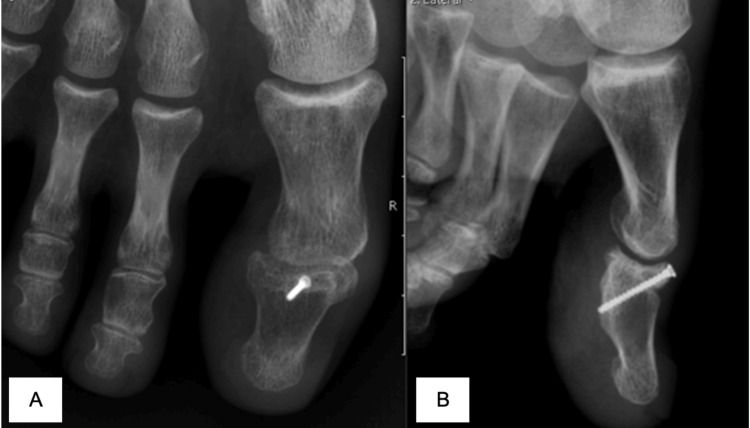
Front (A) and lateral (B) views of the interphalangeal (IP) joint three months post-op.

The three-month follow-up image showed complete bone healing and good restitution of the IP articular surface and congruency (Fig. 4). Active range of motion was also restored at three months, and she returned to all daily activities and sports. 

## Discussion

Unlike mallet toe of the lesser toe fingers, a traumatic mallet deformity of the hallux is rare, and we think it is probably misdiagnosed. High suspicion is important in order to request a proper, strict lateral view of the IP joint of the hallux. This view is essential to identify the lesion and, even more importantly, to choose the treatment - it will inform the size of the bone fragment and the congruency of the IP joint. Sometimes, in case of doubt about the EHL tendon function, ultrasound and magnetic resonance imaging (MRI) can also be requested to help decide the treatment [5,6].

Toe fractures are almost always treated conservatively [7], but since IP joint plantar flexion is essential for propulsion and a correct biomechanical function of the foot, care should be taken when treating these lesions [8].

Mallet hallux injury has been rarely reported in the literature; to our knowledge, only 12 cases have been described [3-13].

The first two cases ever reported were treated conservatively with a dorsal splint for eight weeks or a rigid soled sandal [3,9]. Like in our first case, conservative treatment can be successful and have good outcomes, even similar to those achieved by surgical treatment. Nevertheless, having a splint for many weeks can be hard and have low patient compliance [3,4]. We advocate that conservative treatment is possible and should be considered in selected patients - those with no articular subluxation, minimal displacement, and low demand. Both splinting and a specific shoe can be effective if there is no displacement. 

On the other hand, surgery has its place in mallet hallux treatment. There is also no consensus when it comes to deciding the best type of procedure. As in the mallet thumb, some authors used closed reduction and percutaneous Kirschner wires (K-wires) fixation [4] and also an “extension block pinning” as described by Ishiguro for mallet finger of the hand [10]. This procedure is effective, but it might lead to some stiffness because it prevents early joint mobilization.

Because of joint stiffness, some authors tried alternative procedures without joint K-wire fixation, such as anchor suturing [11]. This procedure can be useful for small bony avulsions, but it can be technically demanding, and it requires an open approach and augmentation suture for a strong reinsertion [12]. Another author opted for transarticular pinning to maintain IP joint position and protect the anchor reinsertion [6].

In our second case, closed reduction was not possible, so open surgical treatment was mandatory. Since our patient had a considerable-sized bone fragment, osteossynthesis was considered and applied using an anatomical reduction and fixation with a 1.7mm single cortex screw under fluoroscopy control. We managed to achieve a strong screw fixation, and we opted for no IP immobilization. 

Tomizuka et al were the only ones describing screw fixation in the previous publications. They opted for an open reduction, osteosynthesis with 2 screws 1.0mm from the hand system for anti-rotational effect and augmentation technique with a non-reabsorble suture, but they also used a transarticular K-wire protecting the main fixation method [13]. We feel they managed to achieve an anatomical reduction and a strong fixation, with the risk of complications of an open reduction might have, but they also had the IP immobilized regarding the possible rigidity. In our case, we proved that a single larger screw is strong enough to achieve good bone healing and EHL function without the need for provisional IP fixation.

## Conclusions

In summary, the management of mallet hallux should be individualized, balancing the simplicity and good outcomes of conservative treatment in selected patients with the need for surgical intervention in cases involving larger fragments, displacement, or joint incongruence. Therapeutic decisions should prioritize restoring hallux function while avoiding stiffness and ensuring stability. Single cortex screw of 1.7mm or similiar diameter is a possible and strong enough fixation method if there is a large bone fragment. 

Given that only a few isolated case reports exist in the literature, further studies and larger case series are needed to better define optimal treatment strategies and long-term outcomes. Because mallet hallux injuries are rare, multicenter studies may be necessary.
